# Extracellular histones, cell-free DNA, or nucleosomes: differences in immunostimulation

**DOI:** 10.1038/cddis.2016.410

**Published:** 2016-12-08

**Authors:** Gerben Marsman, Sacha Zeerleder, Brenda M Luken

**Affiliations:** 1 Department of Immunopathology, Sanquin Research and Landsteiner Laboratory, Academic Medical Center, University of Amsterdam, Amsterdam, The Netherlands; 2 Department of Hematology, Academic Medical Center, Amsterdam, The Netherlands

## Abstract

In inflammation, extensive cell death may occur, which results in the release of chromatin components into the extracellular environment. Individually, the purified chromatin components double stranded (ds)DNA and histones have been demonstrated, both *in vitro* and *in vivo*, to display various immunostimulatory effects, for example, histones induce cytotoxicity and proinflammatory signaling through toll-like receptor (TLR)2 and 4, while DNA induces signaling through TLR9 and intracellular nucleic acid sensing mechanisms. However, DNA and histones are organized in nucleosomes in the nucleus, and evidence suggests that nucleosomes are released as such in inflammation. The cytotoxicity and proinflammatory signaling induced by nucleosomes have not been studied as extensively as the separate effects brought about by histones and dsDNA, and there appear to be some marked differences. Remarkably, little distinction between the different forms in which histones circulate has been made throughout literature. This is partly due to the limitations of existing techniques to differentiate between histones in their free or DNA-bound form. Here we review the current understanding of immunostimulation induced by extracellular histones, dsDNA and nucleosomes, and discuss the importance of techniques that in their detection differentiate between these different chromatin components.

## Facts

- Chromatin components including histones and dsDNA are important DAMPs that induce proinflammatory signaling when released into the extracellular environment.
- Differences exist in the cytotoxicity and proinflammatory signaling induced by free histones and histones as part of nucleosomes.
- Diagnostic tools used to quantify circulating chromatin components often do not discriminate in their detection between histones and nucleosomes
- No clear distinction between circulating histones and nucleosomes is made in the nomenclature of existing literature.

## Open questions

- In what form do histones circulate in inflammatory disease?
- How do the proinflammatory functions of histones compare with those of nucleosomes?
- How to distinguish between free histones and nucleosomes in body fluids?

Various damage-associated molecular patterns (DAMPs) that are released upon cellular damage or cell death are efficient inducers of inflammation. Well known DAMPs are histones and DNA, which reside in the nucleus in the form of nucleosomes. Notably, various immunostimulatory effects including proinflammatory signaling through toll-like receptors (TLRs) and cytotoxicity are initiated when these nuclear DAMPs bind to host cells (see reviews^1, 2, 3^). Certain of these immunostimulatory effects appear to be dictated by the form in which extracellular chromatin molecules are present, that is, histones may either circulate freely or in complex with DNA in the form of a nucleosome. Remarkably, throughout the literature very little distinction between the presence of different forms of histones, DNA, and nucleosomes in clinical samples is made. Moreover, in some research publications the terms histones and nucleosomes are used interchangeably.

In this review we introduce the currently known immunostimulatory functions of cell-free histones and DNA, and compare the separate immunostimulatory effects induced by each, to the effects that are attributable to their complex in the form of extracellular nucleosomes. Furthermore, given that the immunostimulatory effects of these molecules drastically differ, we provide an overview of the current techniques available to detect and quantify cell-free histones, DNA, and nucleosomes in body fluids, and methods to distinguish between these molecules.

## Histone-Induced Inflammation

Histones are highly basic proteins rich in arginine and lysine and are highly conserved amongst species. In humans, an octamer consisting of two dimers of histone H2A and H2B and a tetramer of histone H3 and H4 forms a core around which 147 bp of DNA is wrapped ±1.67 times. The formed complex is referred to as a nucleosome.^4^ The nucleosome structural organization plays an essential role in regulating gene transcription and facilitates efficient higher-order chromatin compaction. The linker histone H1 resides at the stretch of linker DNA that connects two nucleosomes and is essential in regulating chromatin compaction and transcriptional access to the nucleosome.^5^ Histones are widely recognized to bear important proinflammatory functions upon their release from the nucleus into the extracellular environment.^6, 7^

In 2009, Xu *et al.* demonstrated that intravenous injection of histones in mice was lethal within minutes, whilst anti-histone antibodies were found to reduce mortality in lipopolysaccharide (LPS), TNF-*α*, and cecal ligation and puncture models of sepsis.^8^ *In vitro*, it was shown that histones were cytotoxic when added to cultured endothelial cells. In a follow-up study, the authors demonstrated that, in addition to histone-induced cytotoxicity, the injection of sublethal doses of histones in mice resulted in high levels of TNF-*α*, IL-6, and IL-10, which did not occur when TLR4 knock-out (KO) mice were used, whilst the immunostimulatory effect remained in TLR2 KO mice.^9^ In addition, it was shown that histones induce signaling via both TLR4 and TLR2 through the use of specific TLR-transfected HEK cells. Thereafter, Allam *et al.* demonstrated that histones were cytotoxic to renal endothelial cells and tubular epithelial cells *in vitro*, stimulated bone marrow-derived dendritic cells (BMDCs) in a TLR2 and 4 dependent manner, and also induced TLR2 and 4 dependent inflammation *in vivo.*^10^

In addition to histone-induced immunostimulatory signaling via TLR2 and TLR4, Huang *et al.*^11^ demonstrated that TLR9 KO mice were protected from histone-mediated ischemia/reperfusion (I/R) injury. The authors deduced that the exogenous histones may have acted as a cofactor that amplified the TLR9-mediated signaling brought about by endogenous circulating DNA released from dying cells, although direct evidence for this proposed combined role of DNA and histones was not presented in the *in vivo* model. It is important to note that, when investigating the role of TLR9, the translation from mice to men is troublesome. In mice TLR9 is found in macrophages, myeloid DCs, activated T cells, plasmacytoid DCs, B cells, and neutrophils, while in humans, TLR9 expression is limited to plasmacytoid DCs, B cells, and neutrophils. This results in a radically different inflammatory response towards TLR9 agonists in mice compared with humans,^12^ which complicates nuclear DAMP research in animal models.

Another observation that further supports the induction of inflammation by histones was reported by Abrams *et al.,*^13^ who found that neutrophils that were incubated with purified histones released MPO and were activated to form neutrophil extracellular traps (NETs). However, in this process the involvement of TLRs was not investigated.

To understand the mechanisms involved in histone-induced cytotoxicity, several observations reported in the literature provide insight. FITC-labeled histones were shown to bind to the surface of cultured EA.hy926 endothelial cells and subsequently induced an influx of Ca^2+^, which resulted in cell lysis.^13^ Likely, the affinity of histones for phosphodiester bonds does not only ensure their avid binding to DNA but also to the phosphodiester bonds of phospholipids, resulting in the integration of histones into the plasma membrane. Given that histones are strongly conserved throughout evolution, it is not surprising that histones from other species are also cytotoxic through their strong positive charge.^14^ In line with the importance of the positive charge of histones in instigating cytotoxicity, it was found that negatively charged heparin was able to bind and neutralize histones, and independent of its anticoagulant properties abrogated the cytotoxic effects of histones and reduced mortality in murine sterile inflammation and sepsis models.^15^

The negatively charged glycocalyx covering the cell surface appears to determine the sensitivity of different cell types to histone-induced cytotoxicity. Chaaban *et al.*^16^ demonstrated that CHO cells deficient in heparan-sulfate or with inhibited hyaluronan production were markedly more sensitive to histone-induced cytotoxicity. This suggests that the glycocalyx serves as a protective layer to prevent histone insertion into the plasma membrane. Moreover, the glycocalyx may regulate other extracellular functions of histones, as it was shown by Mishra *et al.*,^17^ that extracellular histone H1 binds to polysialic acid (PSA) present in the glycocalyx of cerebellar neurons and Schwann cells, and thus influences nervous system development. It is worth noting that in the study by Chaaban *et al.*, histone-mediated cell death was not affected by TLR2 and TLR4 neutralizing antibodies, indicating that indirect immunostimulation via these receptors was not involved in mediating the cytotoxicity of histones in that experimental setting. However, in a study by Ekaney *et al.*,^18^ a neutralizing anti-TLR4 antibody did inhibit histone-induced cytotoxicity of human microvascular endothelial cells. It is tempting to speculate that these differences may depend on the cell lines used and the ability to form a glycocalyx, however, other differences in the experimental setting, the use of a different inhibitory anti-TLR4 antibody, or distinct different mechanisms of cell death may also be involved.

The discrepant results obtained in experiments using neutralizing anti-TLR antibodies may be explained by the findings in two studies on the ability of histones to activate the NLRP3 inflammasome using either LPS-primed BMDCs,^19^ or Kupffer cells from liver ischemia/reperfusion injury.^20^ Inflammasome activation may result in caspase-1 and caspase-11 dependent pyroptotic cell death in certain cell types (see review^21^), although so far the involvement of specific NLRP3 activation has not been linked directly to pyroptosis. Nonetheless, we hypothesize that histones may induce TLR-mediated inflammasome activation and give rise to pyroptotic cell death. Further studies are required to reveal whether this alternative mechanism of histone-induced cell death exists, in addition to cell death induced by plasma membrane integration of histones. Although histone-induced inflammasome activation was observed in TLR4-deficient Kupffer cells, the involvement of other TLRs remains unexplored. Moreover, it is currently unclear what different cell types are able to execute pyroptosis.

**Figure 1:**
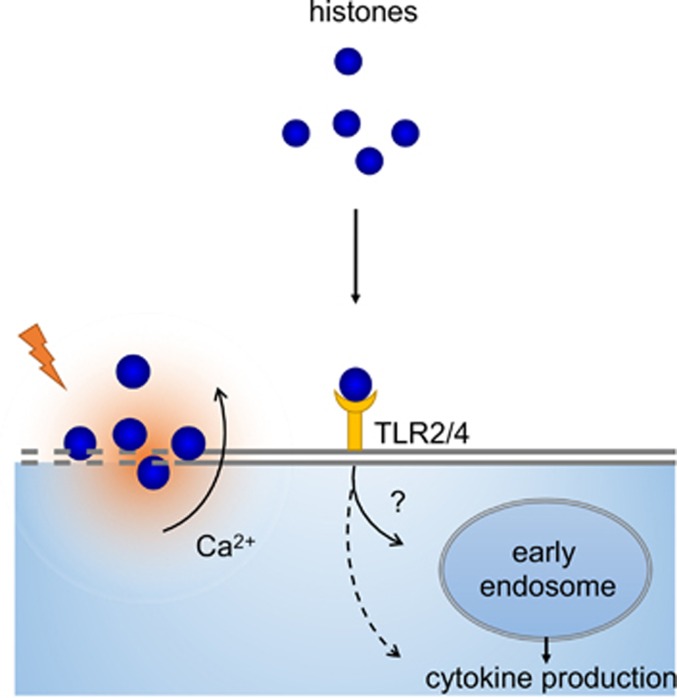
The immunostimulatory effects of histones. Purified histones disturb plasma membrane integrity, which induces a calcium flux, resulting in cellular lysis. In addition, histones have also been shown to signal via TLR2 and 4. It is unclear whether TLR binding of histones induces their uptake and translocation into early endosomes

Thus, histones are unique cytotoxic DAMPs, which elicit both proinflammatory signaling via TLRs and most likely TLR-independent cytotoxicity. As such, histones have a central role in necroinflammation.^22^ For an overview of the immunostimulatory actions of histones, both through TLR signaling and cytotoxicity, see Figure 1.

## Immunostimulatory Actions of Cell Free DNA

Bacterial DNA is a potent immunostimulant as it contains unmethylated CpG motifs that provoke signaling via TLR9.^6^ In contrast, the CpG motifs in vertebrate DNA are mostly methylated.^23^ Indeed, purified vertebrate DNA has repeatedly been found to inadequately activate TLR9.^24, 25^ Furthermore, in a recent study by Bhagirath *et al.*,^26^ a comparison was made between the influences of purified protein-free, and therefore histone-free, nuclear DNA, mitochondrial DNA, and bacterial DNA on human neutrophil viability and IL-6 release. It was found that only mitochondrial and bacterial DNA, which contain unmethylated CpG motifs, increased neutrophil viability as a consequence of their activation. Furthermore, only bacterial DNA induced IL-6 secretion from neutrophils.

Interestingly however, in contrast to purified vertebrate DNA, complexed DNA, either with histones in the form of a nucleosome or DNA in complex with certain DNA-binding proteins, has been demonstrated to induce TLR9-mediated signaling in cultured mouse BMDCs and spleen DCs,^27, 28^ and also *in vivo* in mice.^11^ Several explanations exist for the observed differences in TLR9 stimulation by either purified or complexed DNA. First, since TLR9 in pDCs and B cells is only located in the endosomal compartment, DNA needs to be endocytosed in order to activate TLR9. Purified vertebrate DNA is not easily endocytosed,^29^ but several proteins that bind DNA facilitate its uptake, including C1q,^30^ anti-DNA antibodies,^31^ the receptor for advanced glycation end-products (RAGE),^32^ and histones.^33^ Secondly, in addition to the recognition of unmethylated CpG motifs, the phosphodiester backbone of DNA has been demonstrated to efficiently dimerize TLR9 in solution.^34^ Thus, vertebrate DNA may activate TLR9 in a sequence independent manner.^35, 36^ Finally, in a more recent study, it was shown that TLR9 preferentially recognizes a curved DNA backbone.^34^ We hypothesize that such bending of the DNA backbone occurs in the DNA that wraps nucleosomes, and perhaps also in complexes of DNA with anti-DNA antibodies, or when DNA binds to RAGE. In addition, it has become clear that cell-free DNA may mediate TLR9 independent immunostimulation via cytoplasmic DNA sensing mechanisms such as cyclic GMP-AMP synthase (cGAS), which results in activation of stimulator of interferon genes (STING). Initiation of this pathway by endogenous DNA, but also by dsDNA viruses that have invaded the cell, results in type I interferon secretion, thereby contributing to DNA-mediated immune activation (see review^37^). An important, but so far unaddressed, question is whether nucleosomes that have been taken up by a cell are able to activate the cGAS-STING pathway. The principles of DNA sensing, as well as the determinants required to mount an efficient nucleic acid-driven immune response have recently been reviewed.^38^

**Figure 2:**
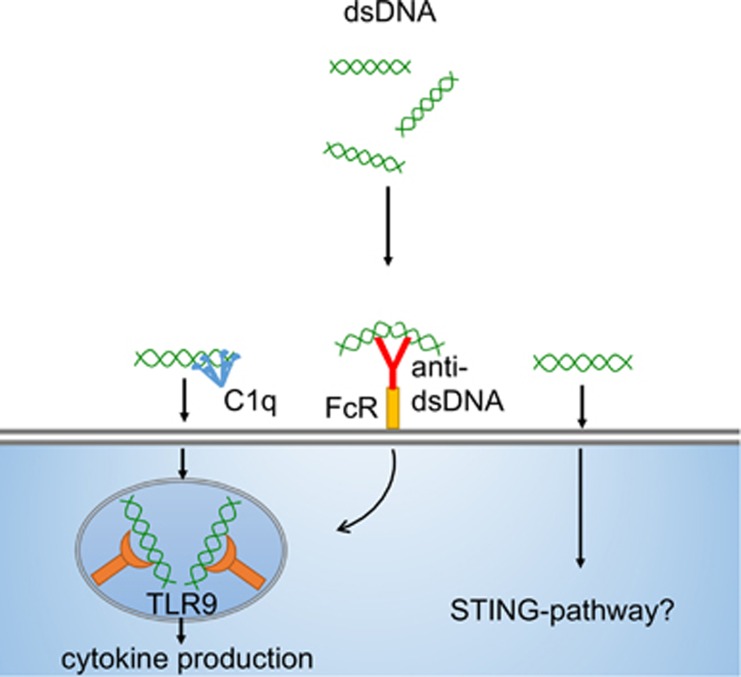
The immunostimulatory effects of dsDNA. Purified DNA is endocytosed and signals via TLR9 or activates cytoplasmic DNA sensing mechanisms. Purified DNA is not easily endocytosed. Several proteins such as C1q, anti-dsDNA antibodies, and histones appear to enhance dsDNA endocytosis. The constraints for TLR9 signaling by dsDNA, including CpG content, the phosphodiester backbone, and DNA curvature, are discussed in the text

Taken together, it is clear that DNA mediates potent immunostimulatory activity, both via TLR9 stimulation as well as via cytoplasmic DNA sensing mechanisms (see Figure 2), and clearly, that the form in which DNA circulates, for example, free or as a nucleosome or immune complex, modulates its immunostimulatory capacity. Furthermore, as discussed above, DNA may serve as a template to enhance TLR2 and 4 signaling instigated by histones.

## The Different Immunostimulatory Effects Induced by Histones and DNA when in the Form of Nucleosomes

A substantial body of evidence suggests that extracellular nucleosomes induce markedly different immunostimulation when compared with free histones and DNA. Rönnefarth *et al.*^39^ have demonstrated that, upon incubation with nucleosomes purified from calf thymus, human neutrophils became activated with CD66b and CD11b upregulation, increased the phagocytosis of added microspheres, and secreted IL-8. Interestingly, in this study, the nucleosome-induced neutrophil activation and recruitment was equally efficient in both WT and TLR2/4 KO mice. In a continuation of the study, it was shown that also TLR9 was dispensable for nucleosome-induced neutrophil activation, even though nucleosomes did induce TLR9 upregulation and increased the response to alternative TLR9 agonists.^40^

Nucleosomes have also been demonstrated to activate human and murine DCs, in a manner that was independent of MyD88.^41^ Given that MyD88 is a signaling protein downstream of all TLRs, with the exception of TLR3, the involvement of TLR2, 4, and 9 in the immunostimulation by nucleosomes was excluded in this study. In contrast, nucleosomes derived from *Plasmodium falciparum* were found to potently stimulate murine DCs in a TLR9-dependent manner.^28^ These results clearly suggest that immune activation by nucleosomes is, in part, determined by the species that the nucleosomes derive from, and that activation may be initiated through distinct receptors in different cell types. To explain the immunostimulatory activity of nucleosomes, the presence of a specific cell-surface receptor that binds nucleosomes has been postulated. Cell-surface proteoglycans have been found to be involved in the binding of nucleosomes to cell surfaces, but the presence of a specific nucleosome receptor has remained elusive.^42, 43, 44, 45^

In addition to differences in inflammatory signaling induced by histones and nucleosomes, the cytotoxic effects ascribed to histones do not appear to apply to nucleosomes. Studies wherein purified nucleosomes were injected in mice to study their clearance lack any mention of cytotoxicity induced by nucleosomes, even at doses of up to 1 mg nucleosomes.^46^ Of note, injection of 1.25 mg of purified histones in mice is lethal within 1 h.^8^ The half-life of injected nucleosomes (2–85 *μ*g) was estimated to be around 4 min, although at higher doses, going up to 1 mg, the clearance of nucleosomes was greatly impaired, suggesting that saturation of the clearance mechanism had been reached. That nucleosomes do not provoke cytotoxicity was confirmed *in vitro* by Abrams *et al.*,^47^ who demonstrated that isolated nucleosomes did not induce cell death of cultured endothelial cells, unless nucleosomes were degraded by brief sonication or upon their incubation with serum. Nonetheless, nucleosomes have been described to induce necrotic cell death specifically in cultured lymphocytes, while DNA and histones did not induce necrosis determined by counting propidium iodide positive, annexin V negative cells.^48^ Given that nucleosomes contain DNA, we speculate that the binding of nucleosomes to lymphocytes, which was also described in that study may, however, have affected the quantification of necrotic cells by propidium iodide staining. Nevertheless, in the same study, it was found that upon injection of nucleosomes in mice, the number of spleen cells, presumably lymphocytes, significantly decreased, while there were no signs that lymphocytes had migrated to other organs. Notably, also in this study, the mice did not display clear signs of inflammation or mortality upon injection with nucleosomes.^48^

**Figure 3:**
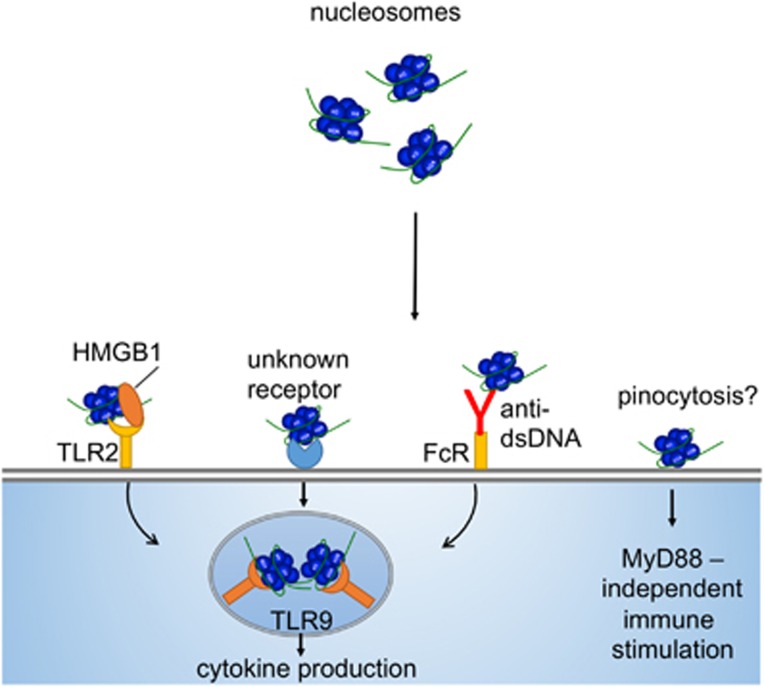
The immunostimulatory effects of nucleosomes. In contrast to purified histones, dsDNA, or mixed preparations, nucleosomes appear to follow additional and different routes of immunostimulation, also depending on the cell type it encounters. Similar to dsDNA, anti-dsDNA antibodies increase the uptake of nucleosomes by phagocytic cells. Moreover, purified nucleosomes with bound HMGB1 mediate immunostimulation of human macrophages via TLR2. In contrast, purified nucleosomes lacking HMGB1 are stimulatory to neutrophils and dendritic cells in a MyD88-independent manner, indicating that stimulation by nucleosomes is also cell-type specific. In contrast to histones, nucleosomes do not appear cytotoxic. Given that nucleosomes were repeatedly found to bind to the plasma membrane, the existence of a nucleosome-specific receptor has been proposed, but this receptor has thus far not been identified. Finally, it is unclear whether nucleosomes that have been taken up by cells are able to stimulate intracellular DNA sensing mechanisms

The potent nuclear-derived DAMP high-mobility group box 1 (HMGB1) may interact with nucleosomes and thus affect immunostimulation as HMGB1 stimulates cells via TLR4 and RAGE.^49^ Nucleosome-HMGB1 complexes have been found in the circulation of SLE patients and were shown to induce the secretion of IL-1*β*, IL-6, IL-10 and TNF-*α* from human macrophages, and the expression of costimulatory molecules in human DCs.^33^ Interestingly, nucleosomes without HMGB1 were not immunostimulatory in this study. Given that HMGB1 was found to strongly bind to nucleosomes in cells that underwent apoptotic, but not necrotic, cell death, the formation and release of nucleosome-HMGB1 complexes may be determined by the type of cell death.^50^ These results suggest that HMGB1 may form a key component of nucleosomes that directly determines their immunostimulatory capacity. Indeed, HMGB1 was shown to bind more avidly to RAGE in the presence of CpG DNA and augmented IFN-γ production by CpG-stimulated human plasmacytoid dendritic cells (pDCs), possibly by enhancing CpG DNA uptake.^51^ Worth noting, in the previously mentioned studies by Ronnefarth *et al.,*^39^ the presence of HMGB1 in their nucleosome preparations was carefully excluded, and neutrophil activation occurred readily. Taken together, the described observations suggest that the prerequisites for immunostimulation by nucleosomes may well be cell-type dependent, and certainly require further investigation. Furthermore, it is still unclear how post-translational modification of histones affects the immunostimulatory potential of nucleosomes. We have summarized the different routes by which extracellular nucleosomes mediate their immunostimulatory effects in a schematic illustration in Figure 3.

## The Origin of Circulating Nucleosomes

Although the obvious source of extracellular nucleosomes are dying or damaged cells, the mechanisms by which nucleosomes are released into the extracellular environment appear multifold and have not been studied in large detail. An important factor in nucleosome release may be the type of cell death that occurs, for example, apoptosis, necrosis, pyroptosis, necroptosis, NETosis, and others. For example, upon apoptosis, caspase-activated DNase (CAD) induces the fragmentation of DNA into oligonucleosomes, a process that does not take place in necrosis. Indeed, nucleosomes have been found on the surface of apoptotic cells,^52^ and are present in apoptotic cell-derived microparticles.^53, 54^ Furthermore, apoptotic cells passively leak nucleosomes, while several plasma proteins such as Factor VII-activating protease (FSAP), which is activated upon contact with late apoptotic or necrotic cells, facilitate the efficient release of chromatin from late apoptotic cells.^55^ In addition to FSAP, Factor H has recently been found to bind nucleosomes and mediate their release from apoptotic cells.^56^ However, this mechanism remains to be validated in full serum or plasma.

In contrast to apoptotic cell death, necrotic cell death does not involve the activity of intracellular nucleases, and chromatin fragmentation is therefore lacking in cells that undergo necrosis. The release of nucleosomes from these cells does not appear to occur passively, but requires additional factors such as circulating nucleases. For example, for FSAP-mediated nucleosome release from necrotic cells, serum DNase activity was required to fragment chromatin before its release into the extracellular environment. This in contrast to FSAP-mediated nucleosome release from late apoptotic cells where, chromatin fragmentation had already occurred and circulating nuclease activity was not required.^57^ Interestingly, it is known that C1q may in its turn increase the activity of serum DNase I, resulting in enhanced necrotic chromatin clearance,^30^ whilst C1q also enhanced the efferocytosis of late apoptotic cells.^58^ The synergistic functions of these plasma factors in facilitating the release and clearance of dead cell chromatin remain to be elucidated.

The mechanisms of chromatin release are starting to unravel. For instance, FSAP efficiently cleaved linker histone H1 in necrotic cells. Since histone H1 mediates the higher order compaction of chromatin (see review^59^), H1 cleavage by FSAP may form a crucial step in the release of chromatin from dying cells. It is clear that chromatin release proceeds in a highly regulated manner and that multiple nucleases, both intracellular as well as extracellular, in combination with various plasma proteins are involved in this regulation. Impaired functionality of these factors, for example, of DNase I has been linked to systemic lupus erythematosus (SLE), whilst the release of chromatin from late apoptotic cells by FSAP has been shown to be inhibited in patients with SLE.^60, 61^

In addition to the release of chromatin from dying non-myeloid cells, activated neutrophils may undergo a form of cell death whereby their chromatin is excreted into the extracellular environment to form so-called NETs.^62^ Notably, this type of cell death has also been found in other cell types, for example, mast cells, basophils, and macrophages (see review^63^). NETs are decorated with neutrophil proteases and have been demonstrated to efficiently trap and kill pathogens.^62^ Interestingly, NETs were shown to be cytotoxic to lung epithelial cells and mouse glomerular endothelial cells *in vitro.*^64, 65^ This effect was, in part, mediated by the histones present in NETs as NETs remained toxic upon DNA digestion, while anti-histone antibodies partially protected cells against NET induced cytotoxicity.

**Figure 4:**
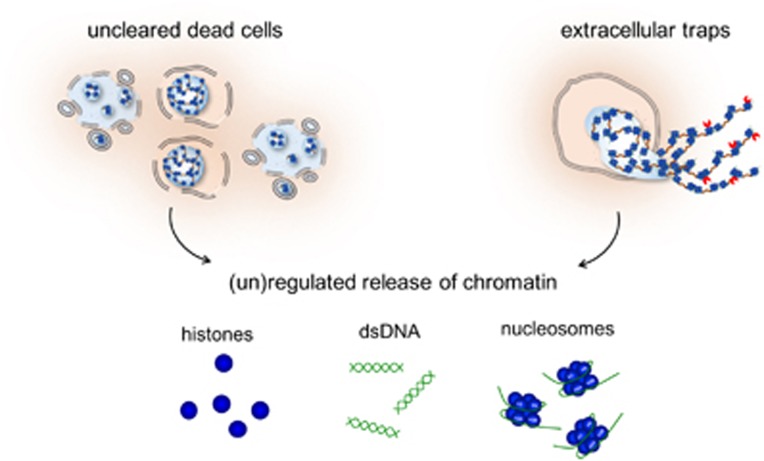
The origin of histones, dsDNA, and nucleosomes. Upon insufficient clearance of dead cells, or the induction of (neutrophil) extracellular traps, chromatin components are released into the extracellular environment. This release may occur passively, but several plasma proteins are known to regulate the release of chromatin (components) from dead cells

Several studies have revealed that, in contrast to NETs, purified nucleosomes are not toxic to cultured endothelial cells. Several explanations may be provided for the apparent differences in the cytotoxicity of NETs and nucleosomes. First, during NETosis, histones are processed by elastase^66^ while peptidylarginine deiminase-4 (PAD4) converts the highly charged arginine residues in histones to more neutral citrulline, which results in a more open chromatin structure.^67^ This may possibly result in an increased exposure of cytotoxic histones when compared with purified unmodified nucleosomes. Secondly, the anti-microbial proteases present in NETs may confer cytotoxicity as well. Indeed, an MPO inhibitor decreased the cytotoxicity of NETs, while an elastase inhibitor had no effect.^64^ Finally, the length of extracellular chromatin, which is much longer in NETs compared with purified nucleosomes, might also contribute to cytotoxicity. In conclusion, extracellular chromatin may derive from different origins ranging from dying non-myeloid cells to NETting neutrophils, and the release of chromatin from these cells appears tightly controlled (Figure 4).

Since the mechanisms that may account for the levels of extracellular chromatin in the circulation are manifold, this raises the question from which cell types the circulating nucleosomes originate. Circulating nucleosome levels are increasingly being used as a marker for NETosis,^68, 69, 70, 71^ but may in fact be derived from various tissues and cell types. Although in several human diseases and murine models nucleosome levels indeed appear to correlate with neutrophil activation as determined by circulating elastase levels,^72^ no assays currently exist that distinguish between NET-derived or non-myeloid cell-derived chromatin. What is currently being used in the literature is an ELISA that specifically detects DNA-MPO complexes which are formed during NETosis.^73, 74^ However, it is still unclear whether the presence of these complexes is specifically linked to NETosis, or whether MPO released upon neutrophil degranulation or from monocytes, may also form complexes with circulating DNA from other cells. Importantly, in PAD4-deficient mice, which are reportedly impaired in NET formation, increased levels of circulating nucleosomes were found upon LPS-challenge, similar to the increase seen in wild-type mice, suggesting that nucleosomes are derived from other cells than NETting neutrophils.^75^ In a different study, Sun *et al.*^76^ studied the origin of circulating DNA through plasma DNA tissue mapping. They found that the majority of circulating DNA in cancer patients and in patients that had undergone a bone marrow or liver transplantation was lymphocyte derived. However, neutrophils contributed significantly to the circulating cell-free DNA level. Future studies employing this tissue-mapping method may help to elucidate the origin of circulating DNA in inflammatory disease.

## Detection of Circulating Histones and Nucleosomes in Disease

Circulating histones and nucleosomes have frequently been found in patients suffering from a wide range of inflammatory conditions, including sepsis,^77^ traumatic injury and surgery,^13, 18, 78, 79^ cerebral stroke,^80^ chronic obstructive pulmonary disease (COPD),^81^ systemic lupus erythematosus,^82^ multiple organ failure,^18^ disseminated intravascular coagulation (DIC),^83^ thrombotic microangiopathies,^84^ sickle cell disease,^68^ paroxysmal nocturnal hemoglobinuria (PNH),^85^ and cancer.^86, 87^ More importantly, circulating levels of nucleosomes correlate with the length of hospital stay in sickle cell disease,^68^ the severity of stroke,^80^ an increased risk of deep vein thrombosis (DVT),^88^ are associated with mortality in children suffering from meningococcal sepsis,^89^ and may serve as a predictive marker for chemotherapy response in cancer patients,^87^ and mortality in trauma injury.^78^

To detect the presence of circulating histones or nucleosomes, several assays are currently in use. The presence of histones in patient samples is easily visualized by means of immunoblot and this assay has been used extensively.^8, 9, 13, 81, 90^ However, when using an immunoblot, it is not possible to distinguish between freely circulating histones, histones bound to DNA, or histones that are part of a nucleosome complex. Similarly, several ELISAs have been developed that quantify specific histone subtypes.^11, 18, 81, 83, 91^ These assays often make use of polyclonal antibodies raised against histones and it is therefore unlikely that the antibodies used in these assays will solely detect free histones. Alternatively, ELISAs have been developed that specifically detect nucleosomes. Our own in-house developed nucleosome ELISA makes use of a monoclonal anti-histone H3 catching antibody and a monoclonal detection antibody that recognizes a structural epitope formed by histone H2A, H2B, and DNA.^92^ This ensures specificity for nucleosomes and we have not observed any cross-reactivity with purified free histones in this assay. A similar ELISA was developed by Roche (Cell Death Detection ELISA^PLUS^) wherein a monoclonal anti-histone antibody is used as a catching antibody in combination with a monoclonal anti-DNA antibody for detection, and this assay has been widely used.^13, 78, 79, 80, 87, 90, 93, 94, 95, 96^

In addition to the ELISA-based measurement of circulating nucleosomes, PCR-based approaches to quantify circulating cell-free DNA have been established. Since all cell-free DNA seems to be circulating in the form of nucleosomes,^97, 98^ the PCR-based quantitation of cell-free DNA appears to deliver very comparable results to a nucleosome ELISA,^99^ although PCR-based approaches require larger volumes of patient material. Moreover, since most DNA seems to circulate in mono- or di-nucleosome fragments,^97, 98^ caution should be taken to design primer sets for PCR that amplify DNA fragments shorter than 147 bp in length.

The nucleosome-specific ELISAs in particular allow for a reliable measurement of circulating chromatin fragments. Regrettably, the absence of assays that specifically detect free histones has sometimes led investigators to assign certain functions to free histones, whereas it is unclear whether these functions may in fact be attributable to nucleosomes instead. In a study by Abrams *et al.*,^13^ immunoblot was used to detect histones and this assay was combined with a nucleosome ELISA to simultaneously detect nucleosomes in samples obtained from severe trauma patients. Initially both histone and nucleosome levels were high in the first hours after trauma, but nucleosome levels dropped after 24 h while histone levels remained elevated for up to 72 h after hospitalization. The blot used to quantify histones was not shown but the density of the bands was assessed using densitometry. It is unclear whether the histones measured at 24 and 72 h were free or whether they remained (partly) complexed with DNA but became undetectable in ELISA.

In the seminal paper of Xu *et al.* on the importance of TLR2 and TLR4 in ‘histone-mediated' immune signaling in ConA challenged mice, nucleosomes were immunoprecipitated from a mouse sample with an antibody against DNA-H2A-H2B. Strikingly, no residual histone H3 was detected upon analysis of the supernatant on immunoblot, which indicates that all histones were present as part of a nucleosome complex and were not circulating freely. This was indeed pointed out by the authors of the study, and it thus appears that not free histones but nucleosomes are responsible for the TLR2- and TLR4-mediated induction of inflammation observed in that study. Since in most *in vitro* studies nucleosomes mainly induced TLR-independent immunostimulation, it is unclear how to interpret the *in vivo* data.

It is possible, however, that the nucleosome preparations used for most *in vitro* studies, which often consist of mono- and di-nucleosomal fragments, are unable to efficiently cross-link TLR2 and TLR4, in contrast to the larger (NET) fragments that may be present locally at inflammatory sites *in vivo.* This is supported by the observation of Xu *et al.*^9^ that histone signaling via TLR2 and TLR4 in TLR-transfected HEK293 cells was enhanced in the presence of exogenously added DNA. Since the immunostimulatory effects of extracellular histones and nucleosomes appear to be different and subject to different clearance/degradation kinetics, this stipulates the importance of specific assays for chromatin components and a clear use of the terms histones and nucleosomes in the literature.

## Conclusions

The important nuclear DAMPs histones and DNA induce immune activation independently of each other in various cell types. Although the strong positive charge of histones contributes to their cytotoxic activity, very little is known about the mechanism and regulation of histone cytotoxicity, and the involvement of TLR receptors. When present in the form of a nucleosome complex, the cytotoxicity of histones appears to be absent. Whether nucleases, either endogenous or exogenous, modulate the release of cytotoxic histones from nucleosomes is a question that remains to be answered. It is becoming increasingly clear that DNA uptake, subsequent TLR9 triggering, and induction of inflammatory pathways, are in fact facilitated by several DNA-binding proteins acting in conjunction, including histones. Remarkably, several other nucleosome-binding proteins such as HMGB1 and RAGE modulate the immunostimulatory activity of both cell-free DNA and nucleosomes. Future studies are needed to address the immunostimulatory activity of the various chromatin components and the complexes they form, as well as the various types of cell death upon which they are released. Such research would greatly benefit from new methods to reliably and specifically detect either histones, nucleosomes, cell-free DNA, and the complexes they form with additional binding partners, for example, HMGB1. In the meantime, care should be taken when selecting a technique and attributing cytotoxic or immunomodulatory signaling effects to either the individual chromatin components histones or DNA, or their complex in the form of nucleosomes.
